# Role of Optical Neuromonitoring in Neonatal Encephalopathy—Current State and Recent Advances

**DOI:** 10.3389/fped.2021.653676

**Published:** 2021-04-09

**Authors:** Kelly Harvey-Jones, Frederic Lange, Ilias Tachtsidis, Nicola J. Robertson, Subhabrata Mitra

**Affiliations:** ^1^Neonatology, EGA Institute for Women's Health, University College London, London, United Kingdom; ^2^Medical Physics and Biomedical Engineering, University College London, London, United Kingdom; ^3^Edinburgh Neuroscience & Centre for Clinical Brain Sciences, The University of Edinburgh, Edinburgh, United Kingdom

**Keywords:** neonatal encephalopathy, Hypoxic ischaemic encephalopathy (HIE), newborn brain injury, neonatal neuromonitoring, NIRS (near infrared spectroscopy), cerebral haemodynamics and oxygenation, cerebral metabolism, cerebral oxygenation

## Abstract

Neonatal encephalopathy (NE) in term and near-term infants is a significant global health problem; the worldwide burden of disease remains high despite the introduction of therapeutic hypothermia. Assessment of injury severity and effective management in the neonatal intensive care unit (NICU) relies on multiple monitoring modalities from systemic to brain-specific. Current neuromonitoring tools provide information utilized for seizure management, injury stratification, and prognostication, whilst systemic monitoring ensures multi-organ dysfunction is recognized early and supported wherever needed. The neuromonitoring technologies currently used in NE however, have limitations in either their availability during the active treatment window or their reliability to prognosticate and stratify injury confidently in the early period following insult. There is therefore a real need for a neuromonitoring tool that provides cot side, early and continuous monitoring of brain health which can reliably stratify injury severity, monitor response to current and emerging treatments, and prognosticate outcome. The clinical use of near-infrared spectroscopy (NIRS) technology has increased in recent years. Research studies within this population have also increased, alongside the development of both instrumentation and signal processing techniques. Increasing use of commercially available cerebral oximeters in the NICU, and the introduction of advanced optical measurements using broadband NIRS (BNIRS), frequency domain NIRS (FDNIRS), and diffuse correlation spectroscopy (DCS) have widened the scope by allowing the direct monitoring of oxygen metabolism and cerebral blood flow, both key to understanding pathophysiological changes and predicting outcome in NE. This review discusses the role of optical neuromonitoring in NE and why this modality may provide the next significant piece of the puzzle toward understanding the real time state of the injured newborn brain.

## Background: Neonatal Encephalopathy

Neonatal encephalopathy (NE) resulting from intrapartum related events is a global health problem accounting for a quarter of neonatal deaths world-wide (1). It is the second most common cause of preventable childhood disability and this has not improved significantly over the past two decades despite hypothermia treatment (2, 3). In high income settings NE affects between 1 and 3/1,000 live births whereas the incidence is up to 10 times higher in mid and low income settings (1–3).

**Figure 1:**
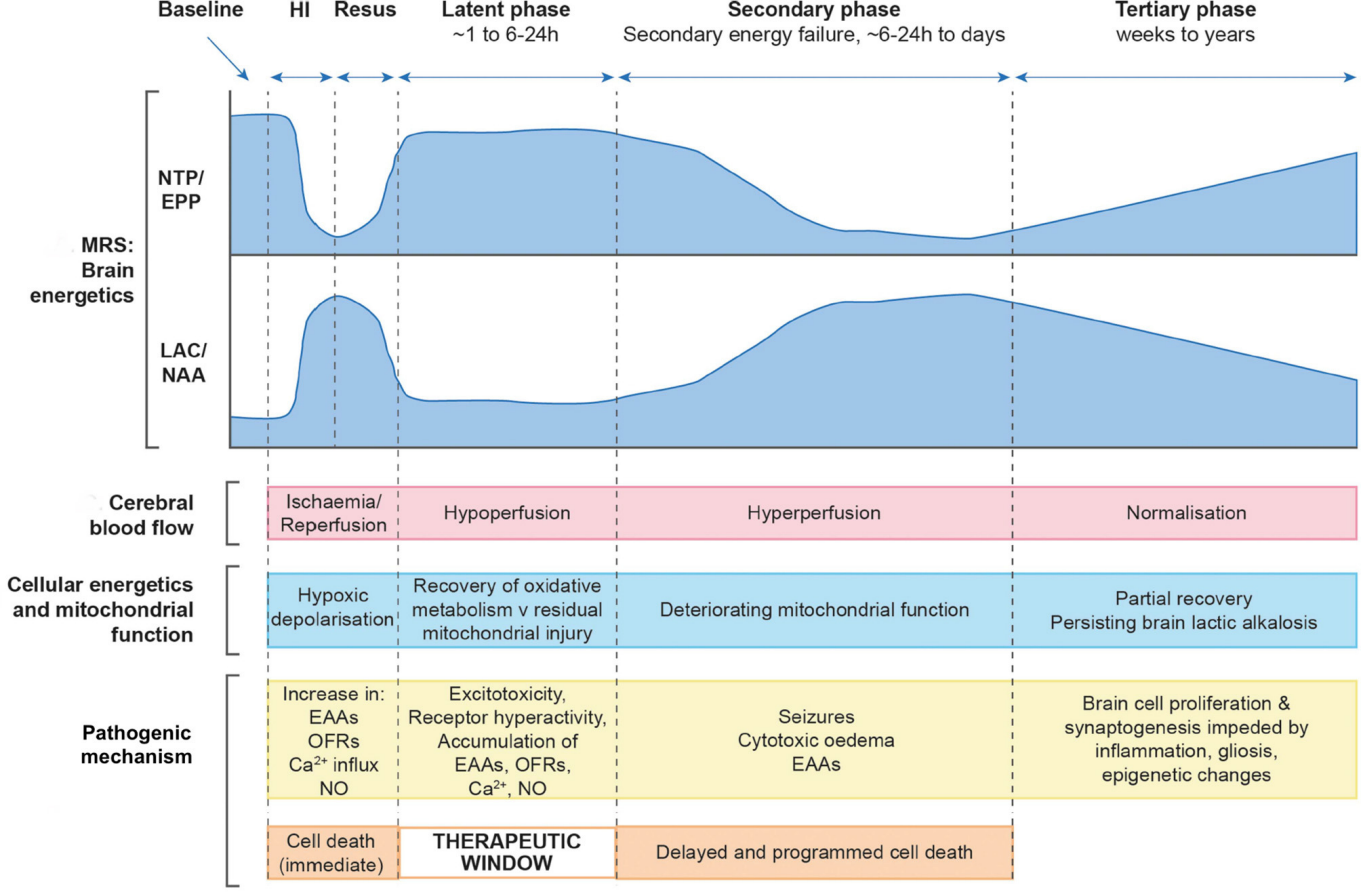
Schematic diagram illustrating the different pathological phases of cerebral injury after hypoxic- ischaemic (HI) insult. Changes in magnetic resonance spectroscopy derived markers of brain energetics (NTP/EPP from ^31^P MRS and Lac/NAA from ^1^H MRS, NTP, nucleotide triphosphate; EPP, exchangeable phosphate pool; Lac, lactate; NAA, N acetyl aspartate) with biphasic pattern of energy failure, cerebral blood flow, cellular energetics and mitochondrial function, pathogenic mechanisms occurring at each phase and cell damage with associated therapeutic window. Image modified from Hassell et al. (9).

Following an intrapartum hypoxic-ischaemic event, the brain suffers significant hemodynamic and metabolic derangements which are dynamic, evolving over a period of hours, days and weeks due to the neurotoxic and neurochemical cascade that ensues (4). Early pre-clinical and clinical studies using phosphorous (^31^P) nuclear magnetic resonance spectroscopy (MRS) described the evolution of secondary energy failure with a reduction in high energy phosphates and a rise in cerebral lactate over hours and days following birth (5, 6). During the initial insult a proportion of cells undergo primary cell death and the neuronal supply of high energy metabolites such as adenosine triphosphate (ATP) is exhausted, also termed “primary energy failure.” Following successful resuscitation the brain enters a latent phase lasting for ~6–24 h which is characterized by the partial recovery of cerebral oxidative metabolism and cerebral blood flow, although a degree of hypoperfusion continues. The brain then enters a period of “secondary energy failure” (SEF) characterized by mitochondrial impairment and cell death with associated cerebral autoregulatory disturbance and brain hyperperfusion. The concept of secondary energy failure (SEF) is a hallmark of NE and the primary target of current therapeutic hypothermia treatment (5–8) (Figure 1).

Therapeutic hypothermia (TH) has been the standard of care for over 10 years for moderate to severe NE in high resource settings like the UK. Animal models post hypoxic-ischaemic (HI) insult have shown that hypothermia reduces energy expenditure and neuronal loss through multiple parallel pathways (8, 10–12). Despite this therapy a significant number of surviving infants with NE suffer moderate to severe disabilities including cerebral palsy, developmental delay, epilepsy, and visual impairment (13, 14). In parallel with the developments in search of new neuroprotective agents to improve outcome, it is essential that neuromonitoring platforms offer accurate and timely information on brain health in order to stratify injury severity, direct clinical care, identify eligible patients for new treatments, and assess their response to them, and ultimately provide vital prognosis of outcome.

## Current Neuromonitoring in the Neonatal Intensive Care Unit (NICU)

**Figure 2:**
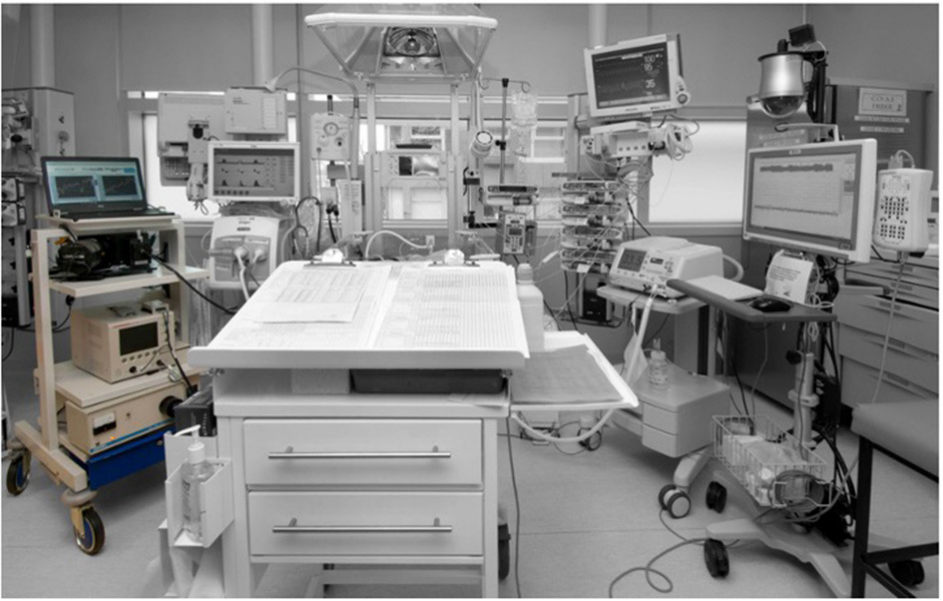
Optimal continuous neuromonitoring in the NICU with video EEG (right), systemic monitoring (right back) and NIRS monitoring (left—a commercial oximeter is used here with a purpose built broadband NIRS system).

Most neonatal centers providing specialist management for infants with NE are currently equipped with essential neuromonitoring tools consisting of some form of neurophysiological monitoring and cranial ultrasound, with or without additional optical monitoring (Figure 2).

### Neurophysiological Monitoring: EEG and aEEG

Amplitude integrated electroencephalograph (aEEG) and electroencephalograph (EEG) have vital roles in the monitoring and management of babies with NE. The information provided assists clinicians in assessing injury severity, tracking progress and determining prognosis with respect to neurological outcome. There are advantages and disadvantages to the use of both neurophysiological tools in NE. aEEG is a filtered and compressed recording of electrocortical activity and displays background amplitude and pattern continuously. Cot side interpretation is typically carried out by neonatologists using pattern recognition and voltage classification (15, 16). A meta-analysis on aEEG use in term infants with NE is available and supports the accurate prognostic ability of background pattern on aEEG to predict neurodevelopmental outcome (17). With the introduction of TH as treatment for NE however, the timing for optimal prognostic accuracy of aEEG is now delayed until 48 h compared with <6 h in the normothermic era (18). Seizure burden is another important and independent prognostic marker in NE (16, 19, 20) and whilst aEEG monitoring is used for seizure detection, a high proportion of short and single electrical seizures may go undetected compared with continuous multi-channel EEG (21–23) which is the gold standard in seizure diagnosis and monitoring of background electrical brain activity (24). A major limitation of continuous multi-channel EEG however, is that probe placement is more complex and interpretation requires prompt and specialist neurophysiology input, something only a few tertiary NICUs can provide as part of their NE management. Most tertiary neonatal units will have access to aEEG and EEG “on demand” but not continuous cot side EEG.

### Cranial Ultrasound

Cranial ultrasound is a simple, non-invasive and cot side imaging modality that is performed serially in NE in an effort to track the progression of injury. Assessment may demonstrate cerebral oedema or in more severe cases increased echogenicity in the basal ganglia and thalamus (BGT). Studies assessing predictive accuracy however, have shown it to be low with a sensitivity of 79% and specificity of 55% for abnormal neurological outcome at 18 months (25).

Transcranial Doppler (TCD) ultrasound is also performed serially in NE to measure the Pourcelot resistance index (RI) (26), calculated from the measurement of peak systolic and end diastolic flow velocity in the anterior cerebral artery. An abnormally low RI in NE suggests cerebral vasodilatation thought to be secondary to cerebral vasoparesis occurring during the phase of secondary energy failure (27, 28). A resistance index of 0.55 or less between 24 and 72 h after birth had a high predictive value for abnormal outcome following NE in the pre-cooling era, (26) however, this has significantly changed following the introduction of TH with the positive predictive value falling from a mean of 84% in normothermia to 60% during TH when measured from 24 h of life (29).

### Magnetic Resonance Imaging and Spectroscopy (MRI/MRS)

Although Magnetic Resonance Imaging (MRI) and spectroscopy (MRS) cannot be performed as part of the continuous neuromonitoring in the NICU, this is currently the optimum neuroimaging technique for detecting perinatally acquired cerebral lesions following NE, and the pattern and severity of these provide a reliable guide to prognosis (30–32). The British Association of Perinatal Medicine's (BAPM) most recent guidance for NE has indicated that all infants undergoing TH should have a brain MRI and proton MRS of basal ganglia-thalamic (BGT) region undertaken between 5 and 14 days after birth (33).

Proton (^1^H) MRS in the neonatal brain has prominent signal peaks related to the presence of N-acetylaspartate (NAA), choline, creatine and lactate (Lac). Metabolite peak area ratios are typically calculated using a voxel positioned in the basal ganglia and thalamic region. An increased lactate and reduced N-acetyl aspartate (high Lac/NAA peak area ratio) on (^1^H) MRS reflects neuronal and mitochondrial injury as well as impaired oxidative metabolism and (^1^H) MRS is highly predictive of outcome in babies who have undergone TH following NE (34, 35). A recent study in newborns undergoing TH for moderate to severe NE noted a threshold of 0.39 Lac/NAA peak area ratio accurately predicted 2 year motor, cognitive and language outcomes (sensitivity and specificity of 100 and 97, 90 and 97, 81 and 97%, respectively) (35). Absolute quantitation of NAA concentration is also reported to have high prognostic value [sensitivity of 1.0 (0.74–1.0) and specificity of 0.97 (0.90–1.0)], but this currently requires a much longer acquisition time and specialist facilities (34).

## Optical Neuromonitoring in Neonatal Encephalopathy

As focus moves toward novel neuroprotective agents targeting underlying secondary and tertiary phases of injury, a very real need persists for a cot side neuromonitoring device in the NICU that will assess injury severity, accurately track the progression of injury from soon after birth, identify babies eligible for novel treatments, and assess their response to those treatments. Ultimately the aim of such a neuromonitoring system would be to offer robust biomarkers of injury in real time that will provide clinicians with vital information regarding severity in order to direct care, and in conjunction with current gold standard techniques such as MRI/MRS accurately inform on neurodevelopmental outcome. Brain monitoring based on optical technologies is relatively cheap, safe, transportable, easy to use and importantly, it allows continuous monitoring at the cot side making it an attractive monitoring system for the dynamic changes occurring in NE. Significant advances have occurred over the past four decades with the development of both near-infrared spectroscopy (NIRS) and diffuse correlation spectroscopy (DCS). Here we present a review of the various optical technologies and findings from their utilization in NE to date (Table 1). We also discuss developments in the instrumentation itself as well as the advances in signal processing techniques used to assess the dynamic relationships between measured parameters. Finally, we discuss current and future research aimed at furthering our ability to more accurately measure the complex pathophysiology taking place in NE and provide answers to key clinical questions in order to improve outcomes.

**Table 1:** Clinical and relevant recent pre-clinical optical studies in Neonatal Encephalopathy: characteristics.

**Optical technology/Device**	**References**	**Study Design**	**No of subjects**	**Assessment aims**	**Optical parameters measured**	**Duration of study**	**Result**
NIRS/Oximetry Radiometer	Van Bel et al. (36)	Observational	31	Cerebral oxygenation	CBV, HbO_2_, HbR, Cytaa_3_	4–6 h	CBV, HbO_2_, HbR and Cytaa_3_ reduced in 1st 12 h in severe outcome group
NIRS/Oximetry NIRO 1000/500	Meek (27)	Observational	27	Cerebral oxygenation	CBV,	1–4 episodes b/w 2–72 h	Increased CBV on 1st day of life is sensitive predictor of adverse outcome
NIRS/Oximetry NIRO 300	Zaramella et al. (37)	Case-control	22	Cerebral oxygenation	TOI, CBV	Day 1 study duration n.r	Increased TOI on day 1 predictive of adverse outcome at 1 yr
NIRS/Oximetry TRS-10	Nakamura et al. (38)	Observational	11	Cerebral oxygenation	ScO_2_,	72 h	Early CBV and ScO_2_ elevations predictive of poor outcome on MRI
NIRS/Oximetry NIRO 200	Ancora et al. (39)	Observational	1	Cerebral oxygenation	TOI	n.r—throughout TH	Early increase in TOI before rewarming. TOI improved with TH and remained stable during rewarming
NIRS/Oximetry INVOS 5100C	Niezen et al. (40)	Retrospective observational	39	Cerebral oxygenation aEEG combined scores	rcSO_2_	96 h	Higher rcSO_2_ from 48 h associated with severely abnormal outcome
NIRS/Oximetry NIRO 200	Ancora et al. (41)	Observational	12	Cerebral oxygenation	TOI	72 h	Higher mean TOI from 12 h is predictive of poor outcome
NIRS/Oximetry INVOS 5100	Arriaga-Redondo et al. (42)	Observational	23	Cerebral oxygenation	rScO_2_	100 h	rScO_2_ >90% during cooling were predictive of poor outcome
NIRS/Oximetry FORE-SIGHT	Wintermark et al. (43)	Observational	7	Cerebral oxygenation Hemodynamics	SctO_2_	84 h	SctO_2_ increased day 1–2 in all newborns. Higher values were predictive of brain injury
NIRS/Oximetry FORE-SIGHT	Peng et al. (44)	Observational	18	Cerebral oxygenation	rSO_2_	79 h	rSO_2_ consistently higher and significantly higher on day 1, in newborns who developed brain injury on MRI
NIRS/Oximetry INVOS	Jain et al. (45)	Prospective observational	21	Cerebral oxygenation	CrSO_2_	48 h	Higher absolute CrSO_2_ during TH correlates with poor outcome on MRI and neurodevelopmental
NIRS/Oximetry INVOS 5100C	Szakmar et al. (46)	Retrospective observational	49	Cerebral oxygenation	CrSO_2_	>72 h	Higher CrSO_2_ at rewarming and from day 1–2 predictive of injury on MRI
NIRS/Oximetry NIRO 200	Massaro et al. (47)	Observational	36	Autoregulation	PPI	84 h	Higher PPI in both hemispheres and high gain in right hemisphere were associated with poor outcome
NIRS/Oximetry NIRO 200	Govindan et al. (48)	Observational	4	Autoregulation	HbD, MABP, PPI	n.r	Modified coherence estimation approach identified to measure assoc between HbD and MABP
NIRS/Oximetry INVOS	Howlett et al. (49)	Observational	24	Autoregulation	HbT, MABP HVx, and MAP_opt_	84 h	Longer time spent and greater deviation below MAPopt during rewarming predicted injury severity on MRI
NIRS/Oximetry INVOS 5100	Burton et al. (50)	Observational	19	Autoregulation	HbT, MABP HVx, and MAP_opt_	84 h	Higher MAPopt, greater time spent and deviation below MAPopt during rewarming found in adverse group at 2 yr
NIRS/Oximetry INVOS 5100	Lee et al. (51)	Prospective observational	64	Autoregulation		90 h	BP deviation from optimal vasoreactivity assoc with injury on MRI
NIRS/Oximetry INVOS	Tekes et al. (52)	Observational	27	Autoregulation	MABP, MAPopt	n.r	MABP deviation below MAPopt during TH and rewarming assoc with lower ADC scalers in PLIC, PP, PCS
NIRS/Oximetry INVOS 4100-5100	Chalak et al. (53)	Observational	10	Autoregulation	SctO_2_ with MABP	72 h	Multiple time scale correlations between oscillations in MABP and SctO_2_
NIRS/Oximetry INVOS 4100-5100	Tian et al. (54)	Observational	9	Autoregulation		72 h	Both in-phase and anti-phase coherence were related to worse outcome
NIRS/Oximetry INVOS 4100	Toet et al. (55)	Observational	18	Cerebral oxygenation Metabolism	rSO_2_, FTOE	48 h	From 24 h, rSO_2_ values increased to supranormal values and significantly higher in adverse outcome group
NIRS/Oximetry INVOS 4100-5100	Lemmers et al. (56)	Observational	39	Cerebral oxygenation Metabolism	rScO_2_, cFTOE	84 h	Higher rScO_2_ values were associated with adverse outcome
NIRS/Oximetry INVOS 5100C	Goeral et al. (57)	Prospective observational	32	Cerebral oxygenation Combined NIRS/EEG scores	aEEG, NIRS, BP	102 h	Combined score of BP, aEEG, and NIRS increased the accuracy of early outcome prediction
NIRS/Oximetry NIRO 200	Govindan et al. (58)	Observational	4	Neurovascular coupling	aEEG	n.r	Surviving infants revealed emergence of NVC during TH
NIRS/Oximetry INVOS 4100-5100	Chalak et al. (59)	Observational	10	Cerebral oxygenation Neurovascular coupling	SctO_2_	60 ± 6 h	NVC significantly decreased in encephalopathic newborns and significantly lower in adverse outcome group
Broadband NIRS In-house built	Bainbridge et al. (60)	Prospective observational	24	Metabolism	Δ[oxCCO]	48 h	Lowered Δ[oxCCO] and NTP/epp 1 h post-HI and slower recovery of Δ[oxCCO] to 1 h predicted adverse outcome
Broadband NIRS In-house built	Kaynezhad et al. (61)	Prospective observational	27	Metabolism Cerebral oxygenation Perfusion	HbO_2_, HHb, HbT, Δ[oxCCO]RF (recovery fraction)	48 h	Δ[oxCCO]- RF cut-off threshold of 79% within 30 min of HI predicted injury severity based on Lac/NAA
Broadband NIRS In-house built	Bale et al. (62)	Observational	6	Metabolism	HbD, HbT oxCCO	Up to 5 d	HbD and oxCCO consistently decreased during desaturations events in unfavorable outcome group
Broadband NIRS In-house built	Bale et al. (63)	Prospective observational	11	Metabolic reactivity	oxCCO,	3 h on D3 of TH	Strong relationship between oxCCO and systemic variables indicated severe injury
Broadband NIRS In-house built	Bale et al. (64)	Prospective observational	50	Metabolism Cerebral oxygenation, Perfusion	HbT, HbD, oxCCO	Up to d4	Strong correlation between oxCCO and oxygenation during spontaneous desaturation episodes was assoc with unfavorable outcome
Broadband NIRS In-house built	Mitra et al. (65)	Prospective observational	14	Metabolism Cerebral oxygenation	HbD, oxCCO	14 h	R/ship between metabolism and oxygenation became impaired with rising Lac/NAA
Broadband NIRS In-house built	Mitra et al. (66)	Prospective observational	14	Metabolism Cerebral oxygenation	HbD, oxCCO	12.5 h	Significant difference between brain oxygenation and metabolism in those with mild and mod-severe EEG abnormalities
Broadband NIRS In-house built	Mitra et al. (67)	Prospective observational	23	Metabolic reactivity	Reactivity index oxCCO and MABP	1 h	Pressure passive changes in brain metabolism assoc with injury severity and outcome
FD-NIRS Imagent	Grant et al. (68)	Observational	43	Metabolism	StO_2_, CBV, CMRO_2_	n.r.	CBV and CMRO_2_ were significantly increased in brain injured group
FD NIRS + DCS In-house built	Dehaes et al. (69)	Observational	27	Cerebral hemodynamics and metabolism	CBF_i_, CMRO_2i_	10–16 s 3 times/location	CMRO_2i_ and CBF lower in neonates with NE during TH compared with post TH and controls
BNIRS + DCS In-house built	Rajaram et al. (70)	Prospective observational	8	Cerebral oxygenation, blood flow and metabolism	HbO_2_, HHb, oxCCO, CBF_i_	n.r.	oxCCO exhibited a delayed response to ischemia while CBF and tissue oxygenation (S_t_O_2_) responses were instantaneous

*aEEG, amplitude integrated electroencephalogram; PLIC, posterior limb of internal capsule; PP, putamen and globus pallidus; ADC, apparent diffusion coefficient; MRS, magnetic resonance spectroscopy; MRI, magnetic resonance imaging; NAA, N-acetylaspartate; Lac, lactate; NIRS, near-infrared spectroscopy; DCS, diffuse correlation spectroscopy; CW-NIRS, continuous wave NIRS; FD-NIRS, frequency domain NIRS; BNIRS, broadband NIRS; HbO_2_, oxygenated hemoglobin; HHb, deoxygenated hemoglobin; HbT, total hemoglobin; HbD, hemoglobin difference; Cytaa3, Cytochrome oxidase; CCO, cytochrome C oxidase; oxCCO, oxidation state of CCO TOI, tissue oxygenation index; rSO_2_, regional oxygen saturation; rScO_2_, regional cerebral tissue oxygen saturation; StO_2_, cerebral tissue oxygenation; SctO_2_, regional cerebral oxygen saturation; CrSO_2_, regional oxygen saturation; rTHb, relative total tissue hemoglobin CAR, cerebral autoregulation; MABP, mean arterial blood pressure; PPI, pressure passivity index; CBF, cerebral blood flow; CBV, cerebral blood volume; MAPopt, mean arterial pressure optimal; MWC, moving window correlation; PCr, phosphocreatine; Pi, inorganic phosphate; NTP, nucleotide triphosphate; RF, recovery fraction; FTOE, fractional tissue oxygen extraction; NVC, neurovascular coupling; CBFi, cerebral blood flow index*.

### Near-Infrared Spectroscopy: Basic Principles

Near-infrared spectroscopy (NIRS) utilizes the relative transparency of biological tissue to light in the near infrared region of 700–1,000 nm. This relative transparency allows us to measure different chromophores, substances which absorb light within the same near infrared (NIR) spectrum. Neonates are well-suited to this form of monitoring due to the relatively lower thickness of their skin and skull bones allowing better NIR light depth penetration of tissues. There are three major oxygen dependent chromophores of interest in clinical NIRS monitoring, oxyhemoglobin (HbO_2_), deoxyemoglobin (HHb), and the redox state of Cytochrome C oxidase (CCO). As all have different absorption spectra within the NIR region it is possible to individually measure their tissue concentration changes.

### NIRS Devices and Measured Parameters

Traditional NIRS devices measure changes in hemoglobin in both its oxygenated (HbO_2_) and deoxygenated (HHb) forms to calculate levels of total hemoglobin (HbT=HbO_2_ + HHb) and hemoglobin difference (HbD= HbO_2_-HHb). Total hemoglobin and hemoglobin difference serve as proxy markers for cerebral blood volume (CBV) and cerebral oxygen saturation, respectively. Hemoglobin difference has also been utilized as a proxy marker for cerebral blood flow (CBF) and has been shown to be a reliable surrogate in previous studies (71, 72). Commercially available cerebral oximeters provide a further NIRS parameter called the absolute tissue oxygen saturation or cerebral oxygenation, with manufacturers using a variety of nomenclature for this parameter (StO_2_, TOI, rSO_2_, rScO_2_, SctO_2_, CrSO_2_). For consistency we will refer to this parameter throughout as the absolute tissue oxygen saturation. Using manufacturer specific algorithms this is delivered as an absolute percentage, calculated as a ratio of oxyhemoglobin (HbO_2_) to total hemoglobin (HbT), averages are calculated in the arterial and venous compartments in the measured tissue and finally weighted by their estimated arterial/venous volume ratio. These ratios are impossible to determine precisely and are most certainly dynamic with proportions likely to vary over time, between different subjects and depending on different disease states (73). For this reason approximations are used in manufacturer algorithms. Beyond commercially available NIRS brain oximetry measurements, other NIRS techniques can also be used to quantify oxidative metabolism and microvascular perfusion, to be discussed in later sections.

There are three main modes of NIRS technology utilized within both commercial and purpose-built platforms. Continuous Wave (CW-NIRS) is the earliest and most common. Using a few (2–4) discrete wavelengths of continuous light source, CW-NIRS does not differentiate between light attenuation caused by absorption and scattering. Absolute concentrations of individual chromophores are not possible for this reason and instead changes in concentrations are measured against an arbitrary baseline. CW-NIRS commercial brain oximeters that measure absolute tissue oxygen saturation employ multi-distance measurements and algorithms such as spatially resolved spectroscopy (SRS) to scale the concentration measurements of hemoglobin and obtain an absolute tissue oxygen saturation value. Frequency domain (FD-NIRS) mode modulates light at a particular frequency and the attenuation of this light and frequency phase shift is measured. Phase shift observations relate to tissue scattering allowing the differentiation from absorption and therefore measurement of absolute chromophore concentrations. This gives FD-NIRS a theoretical advantage with more consistent quantitative measurements. Time-domain (TD-NIRS) is arguably the most complex technology and uses ultrashort pulses of light, measuring the time of flight through tissue with a photon counting device. This mode enables the absolute measurement of chromophores also, through differentiation of scattering and absorption. TD-NIRS instrumentation provides superior depth penetration however, is a complex and costly technology. Of importance to any monitoring technology is high reproducibility and precision of the measured parameters. Several groups have investigated the precision of NIRS-based tissue oximeters within the neonatal population (74–79). Results of this work remain varied with groups finding both high (74) and low (76) levels of precision for the measurement of various NIRS parameters in neonatal cohorts. More recent studies however, have shown promising results with higher precision achieved by ascertaining high tissue homogeneity for each measurement (79) and by accounting for the significant contribution made by systemic fluctuations and attempting to correct for these (78).

## NIRS Monitoring in Neonatal Encephalopathy

NIRS monitoring has been used widely in both pre-clinical and clinical research studies following perinatal insult. Most studies to date have utilized forms of commercial cerebral oximeters and have provided important information regarding changes in cerebral tissue oxygen saturation and hemodynamics. Cerebral oximeters are also becoming increasingly popular on neonatal units amongst clinicians due to their ease of use and their ability to produce a single absolute measurement of brain tissue oxygen saturation for comparison at the cot side. More recently, there has been renewed interest and further development in broadband NIRS (BNIRS), an extension of CW-NIRS which allows us to capture the full NIR optical spectra of brain tissue and more directly measure the intracellular oxidative metabolic state. FD-NIRS has also been utilized in recent studies in both NE (68) and the preterm population (80) for its ability to provide estimates of relative changes in the cerebral metabolic rate of oxygen (rCMRO_2_), a derived NIRS marker of metabolic activity. FDNIRS has since been used in combination with another optical technology, diffuse correlation spectroscopy (DCS), which directly measures microvascular blood flow to more directly calculate the metabolic parameter CMRO_2i_ in NE (69). The advancement of signal processing techniques occurring in parallel to the technology developments has meant that disturbances in dynamic relationships such as those involved in cerebral autoregulation and metabolic reactivity can be better characterized and monitored, improving our understanding of this disease and bringing us closer to developing robust real-time cot side biomarkers of injury.

### Monitoring Absolute Cerebral Tissue Oxygen Saturation

**Figure 3:**
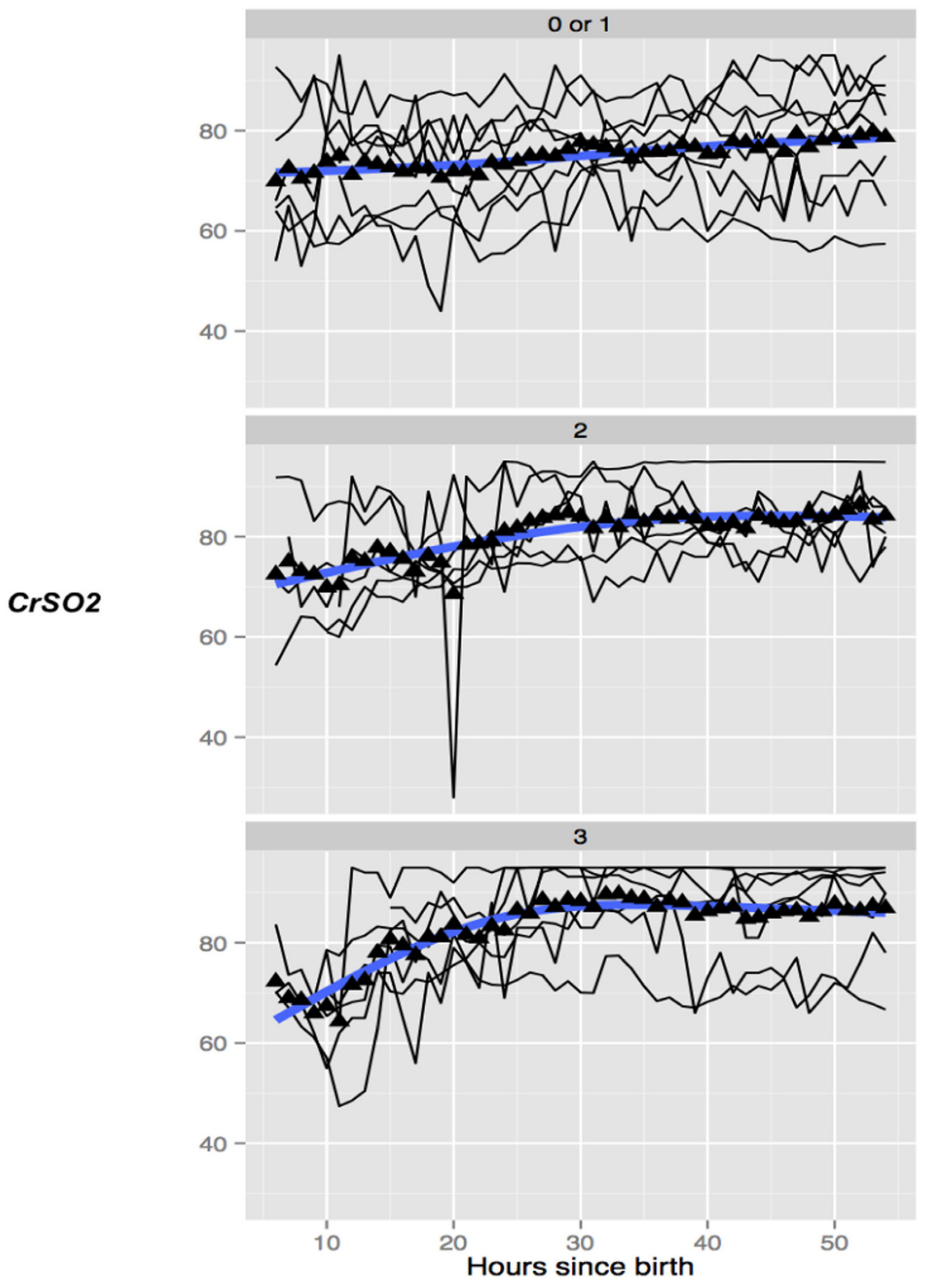
Trend of absolute cerebral tissue oxygen saturation (CrSO_2_) values in 3 groups of infants undergoing TH with none/mild (top), moderate (middle), and severe (bottom) grade of overall MRI injury. The mean values in the group are marked with a solid triangle while the individual infants are represented by thin black lines. CrSO_2_ in infants with no or mild MRI injury (top) remains relatively stable while in infants with moderate (middle) and severe (bottom) CrSO_2_ shows a trend toward an early increase. Reproduced with permission, Jain et al. (45).

In healthy term infants, trends in absolute cerebral tissue oxygen saturation show levels to be lowest at birth (between 40 and 56%) (81–86), rising gradually over the first 24 h following birth to ~78% (±7.9%) (87). Over the next few weeks levels stabilize between 55 and 85% (88–90). Several studies reviewed trends in absolute cerebral tissue oxygen saturation and hemodynamics following perinatal insult in both the pre-TH and TH era. Van Bel et al. found that pre-TH, CBV and absolute cerebral tissue oxygen saturation were observed to fall 12 h after birth in more severely asphyxiated infants with these parameters stabilizing by 12–24 h (36). Higher CBV and CBF values on the 1st day of life in infants pre-TH were associated with more severe injury at neurodevelopmental follow up (27, 37). This finding was supported by a further study looking at both cooled and non-cooled infants showing CBV at 6 h after birth and absolute cerebral tissue oxygen saturation by 24 h were significantly higher in infants with adverse outcomes on MRI. By combining both parameters at 24 h, the ability to predict adverse neurological outcome on MRI between 7 and 14 d after birth was significant with a sensitivity and specificity of 100% (38). Subsequently, several clinical studies in NE over the period of TH and re-warming have found significant differences in the absolute cerebral tissue oxygen saturation values between favorable and adverse outcome groups as determined by MRI or neurodevelopmental follow up (39–46). Cerebral oximetry monitoring commenced within 6 h after birth in one study of cooled infants found absolute cerebral tissue oxygen saturation (rSO_2_) fell following the onset of cooling in all infants followed by a gradual rise then plateau by ~24 h. rSO_2_ significantly increased from day 1 to day 2 of life in both outcome groups but was consistently higher throughout the 72 h hypothermia treatment in asphyxiated newborns developing later brain injury when compared to those with favorable outcomes. This difference between groups was especially prominent during the first 10 h of hypothermia treatment suggesting an early predictive ability for later outcome using this biomarker (44). Further studies during TH have found that higher absolute cerebral tissue oxygen saturation (CrSO_2_, SctO_2_) between 24 and 36h after birth (43, 45, 46) and rewarming (46) predicted adverse neurological outcome as measured by moderate to severe injury on MRI (Figure 3) (45). Rate of rise of CrSO_2_ from baseline until 36 h was also significant in differentiating between outcome groups on MRI (45).

The observed findings of this combined work reflect the known pathophysiological changes occurring in NE. The relative increase in absolute cerebral tissue oxygen saturation can be attributed to profound mitochondrial dysfunction during secondary energy failure leading to reduced oxygen utilization and therefore higher cerebral tissue saturations, the degree of which relates to injury severity. The elevated CBV within the first 24 h following injury reflects the hemodynamic disturbances of vasoparesis and hyperperfusion which also underpin the disease.

### Monitoring Cerebral Autoregulation

Cerebral autoregulation (CAR) refers to the physiological ability of the body to maintain steady cerebral perfusion in response to changes in cerebral perfusion pressure (CPP) by modulating vascular resistance through arteriolar caliber (91). As a consequence of a hypoxic-ischaemic perinatal insult, vasoparesis occurs leading to impaired pressure autoregulation and irregularities in cerebral blood flow. This pressure passivity, or the inability to effectively regulate cerebral blood flow (CBF) in the setting of changes in systemic blood pressure has been well-documented in both animal and human studies and is implicated in the pathogenesis of secondary injury in NE (27, 28). Measuring CPP in neonates is difficult and so mean arterial blood pressure (MABP) has become a necessary surrogate. Numerous studies using NIRS monitoring have sought to characterize the autoregulatory disturbances occurring in encephalopathic newborns using MABP and proxy markers of cerebral perfusion. These are summarized in Table 2. As the relationship between variables is a complex and dynamic one, signal processing techniques used to define them have also seen steady advances in both time and frequency domain analysis techniques. Studies have compared NIRS measurements with injury outcomes to demonstrate how the degree of cerebral autoregulatory disturbance, or pressure passivity, often demonstrated by a change in reactivity limits as opposed to the loss of CAR altogether, can assist in predicting adverse outcomes. It is hypothesized that these unregulated changes in cerebral perfusion can cause uncoupling with cerebral energy metabolism resulting in further potential injury during secondary energy failure.

**Table 2:** Optical studies investigating autoregulatory disturbances in NE.

**Optical technology/Device**	**References**	**No. of subjects**	**Parameters measured**	**Reactivity Index**	**Methodology for assessing autoregulation**	**Results**
Cerebral oximeter NIRO 200	Massaro et al. (47)	36	MABP vs. HbD (CBF)	Pressure passivity index (PPI) and gain	Spectral coherence	Higher PPI and gain (cerebral pressure passivity) was predictive of adverse outcome
Cerebral oximeter NIRO 200	Govindan et al. (48)	4	MABP vs. HbD (CBF)	Pressure passivity index (PPI) and gain	Modified spectral coherence	Higher PPI associated with death or severe MRI abnormalities
Cerebral oximeter INVOS	Howlett et al. (49)	24	MABP vs. rTHb	HVx, MAPopt	Moving correlation coefficient	Greater time spent with MABP below MAPopt during rewarming associated with greater injury on MRI
Cerebral oximeter INVOS 5100	Burton et al. (50)	19	MABP vs. rTHb	HvX, MAPopt	Moving correlation coefficient	Higher calculated MAPopt and longer time spent below during rewarming assoc with worse neurodevelopmental outcomes
Cerebral oximeter INVOS 5100	Lee et al. (51)	64	MABP vs. rTHb	HVx, MAPopt	Moving correlation coefficient	Greatest deviation and longest duration below MAPopt associated with MRI abnormalities
Cerebral oximeter INVOS	Tekes et al. (52)	27	MABP vs. rTHb	HVx, MAPopt	Moving correlation coefficient	Greater deviation below MAPopt associated with restricted diffusion (low ADC values) on MRI
Cerebral oximeter INVOS 4100-5100	Chalak et al. (53)	10	MABP vs. SctO_2_	–	Moving time window correlation	In infants with adverse outcome, both in-phase and anti-phase correlations between MABP and StO_2_ were seen
Cerebral oximeter INVOS 4100-5100	Tian et al. (54)	9	MABP vs. SctO_2_	–	Wavelet coherence analysis	Increased coherence or cerebral pressure passivity predicted adverse outcomes

*MABP, mean arterial blood pressure; HbD, hemoglobin difference; CBF, cerebral blood flow; PPI, pressure passivity index; rTHb, relative total tissue hemoglobin concentration; HVx, hemoglobin volume reactivity index; MAPopt, optimal MABP reactivity range; SctO_2_, regional cerebral oxygen saturation*.

Using continuous NIRS and systemic monitoring Massaro et al. used spectral coherence to compare changes in MABP and HbD, a proxy for CBF, over time. An increase in coherence between these parameters demonstrated a pressure passive cerebral circulation, or disturbed autoregulation. They defined both a pressure passivity index (PPI) and gain to quantify the duration and magnitude, respectively of the passivity and found a higher PPI and gain, therefore higher duration and magnitude of cerebral pressure passivity, were predictive of adverse outcomes (death or detectable brain injury by MRI, P 0.001) (47). Govindan et al. demonstrated similar findings with a modified approach to the coherence analysis (48). Howlett et al. also conducted a NIRS-MABP study looking at changes in cerebral blood volume (CBV). They investigated a reactivity index (HVx) between CBV, measured as relative total tissue hemoglobin concentration (rTHb), and MABP to identify an optimal range of MABP (within 5 mmHg) in which the greatest pressure reactivity (MAPopt) of the cerebral vasculature was noted. A greater severity of brain injury on MRI was associated with more time spent with MABP below this MAPopt during rewarming while those with little to no injury spent more time with MABP within or above the MAPopt range (49). Further studies using the HVx reactivity index found similar results when comparing with 2-year neurodevelopmental outcomes; those infants with lower cognitive scores were found to have higher calculated MAPopt and spent more time below this range during rewarming. In contrast, infants with more favorable outcomes and higher cognitive scores had MABP with greater deviation above their calculated MAPopt (50). These results suggest a shift in the limits of optimal autoregulatory function following injury. Results of a larger study by Lee et al. during TH and rewarming comparing reactivity indices to MRI outcomes further support these findings. Infants with greatest deviation and longest time spent below their MAPopt had more pronounced injury in the white matter and paracentral gyri on MRI (51). Greater deviation below the MAPopt was also associated with restricted diffusion (low ADC values) on MRI in the posterior centrum semiovale and the posterior limb of the internal capsule (52). Chalak et al. were able to investigate more intricately the dynamic properties of cerebral autoregulation following insult. In a pilot study of infants undergoing TH and rewarming using a moving time window correlation (MWC), the relationship between MABP and absolute cerebral tissue oxygen saturation (SctO_2_) was quantified across multiple time scales. In infants with adverse outcomes at neurodevelopmental follow-up, large unprovoked fluctuations in MABP during TH were observed. Both in-phase and anti-phase correlations between MABP and SctO_2_ were seen at different time scales suggesting that infants with abnormal outcomes had two different patterns of impaired CAR dependent on the time scale studied (53). This was supported by the findings of a larger study by the same group which used wavelet coherence analysis, a time-frequency approach to characterize the same relationship between MABP and SctO_2_ during spontaneous oscillations in both. Cerebral autoregulation in babies with NE was found to be time-scale dependent in nature with spontaneous changes in MABP and SctO_2_ having both in-phase and anti-phase coherence at shorter and longer time scales, respectively. Increased coherence or cerebral pressure passivity was observed in infants with adverse outcomes on both MRI and neurodevelopment at 18–24 months and was often sustained for hours to days. In contrast, all of the neonates with normal clinical outcomes showed non-significant coherence between fluctuations in MABP and SctO_2_ most of the time during hypothermia (54).

These studies demonstrate a timeline in the refinement of data analysis in NIRS monitoring in order to quantify the significance of cerebral autoregulatory disturbance in neonatal encephalopathy and how this predicts injury severity and outcome. The development of NIRS based reactivity indices provides a potential physiological biomarker of injury severity which may help direct care by optimizing hemodynamic management in order to prevent secondary brain injury. Larger cohorts using agreed methods and measurements to quantify the autoregulatory disturbances however, are required before determining the full potential of these biomarkers.

### NIRS Monitoring of the Metabolic State: Broadband NIRS

The development of secondary energy failure in NE and its relationship with the degree of injury and neurodevelopmental outcomes is well-established, indicating the importance of monitoring cerebral mitochondrial metabolism along with changes in perfusion. Multiple studies measuring absolute cerebral tissue oxygen saturation in both pre-TH and TH cohorts demonstrated that raised values particularly from 24 h after birth predicted adverse neurological outcome and increased severity of injury on MRI. These findings are explained by the onset of secondary energy failure which leads to reduced oxygen utilization by mitochondria and therefore higher cerebral tissue oxygen saturations. In order to characterize the metabolic disturbance in NE more accurately, we need to measure this metabolic disturbance directly. This has led to the development of broadband NIRS (BNIRS), a technological extension to the CW-NIRS platform. In addition to traditional hemoglobin chromophores, BNIRS technology allows us to measure changes in the redox state of another chromophore, cytochrome c oxidase (CCO). CCO is the terminal acceptor in the mitochondrial electron transport chain and is responsible for 90% of ATP production. Both the oxidized and reduced forms of CCO are chromophores with different absorption spectra in the NIR region. Accurate measurement of concentration changes in the oxidized form (oxCCO) therefore allows us to directly monitor brain metabolic state at a cellular level. CCO as a chromophore has been of interest in the NIRS community for some time as discussed in the seminal paper by Jobsis (92), however, efforts to accurately monitor concentration changes *in-vivo* proved challenging due to its relatively small concentration compared to that of hemoglobin, which dominates the spectral profile. Significant work was carried out by earlier research groups in the 1980's and 1990's with the development of various algorithms using different wavelength numbers aimed at accurately measuring the cytochrome c oxidase redox state *in-vivo*. An analysis of these algorithms (93) found that a higher number of wavelengths produced more accurate measurements. Several reviews summarizing this early work and its challenges are available (93–95). These challenges have since been resolved by the more recent development of the BNIRS technology. The instrument delivers a large number of wavelengths in the 780–900 nm region of NIR light. By using hundreds of wavelengths and excluding shorter wavelengths <780 nm, we see improved signal to noise ratios and a reduction in cross-talk artifacts between different chromophores, such as from HHb which has a large peak at 760 nm (95). Research groups have then utilized various algorithms, summarized by Matcher et al. (93) as well as the *in-vivo* spectra of the difference between oxidized and reduced CCO to derive a measurement of changes in its oxidation state (oxCCO) (95). Measuring changes in oxCCO therefore allows us to directly measure mitochondrial energy metabolism.

In a pre-clinical study of newborn piglets using BNIRS and (^31^P) MRS following an induced hypoxic-ischaemic injury, significant correlations between brain tissue changes in oxCCO and those of metabolite peak area ratios of phosphocreatine (PCr), inorganic phosphate (P_i_) and nucleotide triphosphate (NTP), used to signal brain metabolic state, were noted during and after insult. The recovery of oxCCO but not HbT or HbD, was highly correlated with (^31^P) ratios and predictive of outcome at 48 h (60). A further pre-clinical experiment by the same group quantified the recovery in brain oxygenation (HbT and HbD) and metabolism (oxCCO) for up to 30 min following a hypoxic-ischaemic insult. The recovery fractions (RF) in BNIRS signals were then compared to (^1^H) MRS derived thalamic Lac/NAA as an established surrogate marker of neurodevelopmental outcome. The oxCCO RF cut-off threshold of 79% within 30 min of HI predicted injury severity with high sensitivity (100%) and specificity (93%) and offered a real-time biomarker of brain injury severity within 30 min following HI insult (61). Bale et al. described the new BNIRS instrument for clinical use (62) and carried out a feasibility study on six encephalopathic newborns. The study monitored NIRS variables for cerebral hemoglobin oxygenation and hemodynamics as well as mitochondrial oxygen utilization via oxCCO with analysis focussed on response to spontaneous oxygen desaturations. Changes in oxCCO during the desaturation events were significantly associated with injury severity (*r* = 0.91, *p* < 0.01) (62). A close correlation between oxCCO concentration changes and systemic variations was able to predict injury on (^1^H) MRS suggesting a higher oxygen dependency of mitochondrial metabolism in cases with unfavorable outcome (63). The relationship between cerebral oxygen delivery and mitochondrial oxidative metabolism also indicated injury severity during TH (64) and rewarming (65, 66). In the study by Mitra et al., the relationship between cerebral mitochondrial metabolism and oxygenation (measured as oxCCO and HbD, respectively) during rewarming became more impaired with rising Lac/NAA on (^1^H) MRS, reflective of injury severity (65). Results suggest that following severe hypoxia-ischaemia and cell death, cerebral metabolism failed to improve in spite of oxygen availability. Mitra et al. went on to develop a metabolic reactivity index using oxCCO and MABP using wavelet semblance (phase relationship) and noted a high semblance at 48 h after birth correlated well with injury severity on MRI imaging, Lac/NAA on (^1^H) MRS and neurodevelopmental outcomes (67).

These studies demonstrate a new potential biomarker of injury with a trend toward increased passivity of mitochondrial activity (changes in oxCCO) in response to systemic variations in the adverse outcome groups. This loss of metabolic reactivity is consistent with known pathophysiological changes of mitochondrial dysfunction and is possibly explained by lower cellular energy reserves in the more injured brain meaning CCO has less capacity to buffer changes in systemic variations (63).

### Other Optical Indicators of Metabolic Disturbance in NE

Some studies have also sought to measure the metabolic changes occurring in neonatal encephalopathy using traditional cerebral oximetry. Two studies in the pre-TH and TH era, respectively (55, 56), measured the derived parameter of fractional tissue oxygen extraction (FTOE). This parameter utilizes the absolute cerebral tissue oxygen saturation (StO_2_, rSO_2_, rScO_2_) and systemic arterial saturations (SaO_2_) to represent the balance between oxygen delivery from systemic arterial supply and oxygen utilization by cerebral tissues through mitochondrial metabolic activity (FTOE= SaO_2_-StO_2_/SaO_2_). An increase in FTOE suggests an increase in oxygen consumption by cerebral tissue (seen as lower absolute cerebral tissue oxygen saturation) and a reduced FTOE would suggest reduced oxygen consumption or metabolic activity relative to oxygen delivery. In a pre-TH study by Toet et al., the rSO_2_ and FTOE remained stable in infants with normal outcomes whilst in those with an adverse outcome the rSO_2_ increased and the FTOE decreased after 24 h (rSO_2_: 65 vs. 84% at 12 and 48 h, respectively) (55). The same group carried out a further study in the TH era and similarly found that in the adverse outcome group using 18 month neurodevelopmental assessment, rScO_2_ reached high values from 24 h after birth onwards and values were significantly higher than in the favorable outcome group at 24, 36, 48, and 84 h postnatally. The mean FTOE value mirrored the patterns of rScO_2_ of both groups and became very low from 24 h of age onward in the adverse outcome group as compared with the favorable outcome group (56).

The cerebral metabolic rate of oxygen consumption (CMRO_2_) is another derived parameter of brain metabolic state. NIRS measurements of changes to oxy and deoxyhemoglobin as well as proxy markers for tissue blood flow combined with arteriovenous systemic oxygen saturations enable investigators to derive estimates for the relative changes in cerebral oxygen metabolism (rCMRO_2_). In a study by Grant et al. investigating a cohort of infants following perinatal insult in the pre-TH era, increased CBV and CMRO_2_ were sensitive markers in detecting evolving neonatal brain injury as evidenced on ultrasound and MRI (68). Another optical monitoring technology, diffuse correlation spectroscopy (DCS) which will be discussed in more detail, enables more direct measurement of microvascular cerebral blood flow (CBF_i_). Hybrid instrumentation combining DCS with FD-NIRS offers the possibility for a more accurate determination of this measurement, CMRO_2i_, and has been utilized so far in a single study of infants with NE undergoing TH (69). Cerebral metabolic rate of oxygen (CMRO_2i_) and CBF (CBF_i_) measurements were significantly lower than post-TH and age-matched controls (69). The difference in findings in the above two studies may be explained by a more severely affected cohort in the study by Grant et al. as well as the finding of a lower CBF_i_ in the latter study, which was measured directly using DCS.

### NIRS Monitoring in Combination With Other Neuromonitoring Tools

Several studies combined NIRS parameters with aEEG data to strengthen their ability to predict outcome in NE (56, 57). Lemmers et al. used the combined score of aEEG and absolute cerebral tissue oxygen saturation to improve the positive predictive ability for neurodevelopmental outcome to 91% from 62% and 67% individually (56), whilst Goeral et al. found similar strength in combining these two modalities, with highest predictive abilities of the combined scores between 18 h and 60 h of TH (57). Govindan et al. demonstrated the feasibility of quantifying neurovascular coupling (NVC), whereby neuronal activation produces hemodynamic changes and vice versa continuously in infants with NE undergoing therapeutic hypothermia. The coupling between simultaneous background EEG and NIRS was quantified by calculating spectral coherence between the two signals (58). Chalak et al. further investigated neurovascular coupling (NVC) using wavelet analysis of the dynamic coherence between aEEG and NIRS measured absolute cerebral tissue oxygen saturation. Significantly decreased NVC coherence was observed in encephalopathic newborns undergoing TH with adverse outcomes at 24 months compared to non-encephalopathic newborns (59).

The advantages of simultaneous NIRS monitoring in conjunction with video EEG in neonatal and pediatric epilepsy is summarized in the paper by Wallois et al. (96). The added information gained by NIRS monitoring of the metabolic and hemodynamic disturbances surrounding seizure activity may have the potential to predict seizures and/or optimize seizure management (96), which is highly advantageous in the NE population given the seizure burden in this disease and its independent contribution to neurological injury (19, 97).

## Diffuse Correlation Spectroscopy

Cerebral blood flow is a vital component of brain health linking metabolic demand to oxygen delivery and in turn, oxygen utilization to by-product clearance. Its dynamic properties are integral to brain autoregulatory functioning and neurovascular coupling so the ability to measure this more directly in NE is highly desirable given the pathophysiology effecting these processes in affected newborns. Diffuse correlation spectroscopy (DCS) is a relatively new optical technology which measures fluctuations in emitted light caused by scatter occurring within moving cells in tissues, predominantly by red blood cells. DCS shares the light penetration advantages of NIRS but as it explicitly measures cell movement, it provides a direct measurement of quantities such as cerebral blood flow (CBF) (98). As the measured temporal fluctuations are directly proportional to the speed of the scatter and the tissue within which it is moving, DCS can be used to directly measure micro-vascular blood flow in the brain by providing a cerebral blood flow index (CBF_i_, cm^2^/s) (99). As the diffusing light is mostly absorbed when traversing large arteries and veins, DCS is most sensitive to the motion of blood cells in the microvasculature (98). Abnormalities in brain perfusion following a hypoxic-ischaemic insult have previously been investigated using proxy markers for large vessel blood flow provided by cerebral oximetry, such as HbD and HbT, representing CBF and CBV, respectively. By measuring local microvascular blood flow directly and continuously, DCS can capture the dynamic nature of autoregulatory disturbances which is a desirable addition to our neuromonitoring potential. The addition of a direct blood flow measurement has also had a significant impact on another all-optical measurement of metabolic state, CMRO_2i_.

As seen with NIRS technology, infants make excellent populations to study with DCS due to thinner extracerebral layers and generally less hair leading to higher observed signal to noise ratio (SNR), excellent reproducibility of CBF_i_ and greater sensitivity to cortical tissue compared to adults (100). None of the alternative technologies currently available for measuring CBF such as positron emission tomography (PET), arterial spin labeling MR (ASL-MR), single photon emission computed tomography (SPECT) or Xenon and dynamic computed tomography (CT), are suitable for monitoring at the cot side in NICU due to their impractical size or use of ionizing radiation. Thus, an opportunity exists for DCS capabilities to measure CBF easily, non-invasively, safely, and continuously at the cot side. Validation studies of CBF_i_ have been carried out with dynamic contrast enhanced TD-NIRS (101) and BNIRS (102) using Indocyanine green (ICG) as a flow tracer, fluorescent microspheres (103) as well as ASL-MR (104). All have shown very good correlation between measurements of cerebral blood flow. DCS monitoring of CBF has been employed in a wide range of research contexts in the neonatal population including various preterm cohorts (89, 102, 105, 106), in post-op cardiac surgery (107) and in neonates and children born with severe congenital heart disease (104). In the preterm cohorts, a combination of FDNIRS with DCS simultaneously measured parameters of absolute cerebral tissue oxygen saturation (StO_2_), CBV and blood flow index (CBF_i_) to determine their relationships with gestational age and chronological age (105). Combining DCS and FD-NIRS parameters, changes in the rCMRO_2_ were also measured serially over the first 6 weeks of preterm life (89) and following functional activation (106). The study by Diop et al. (102) using a combined BNIRS and DCS platform successfully monitored CBF_i_ and CMRO_2_ in a preterm population undergoing Indomethacin treatment for PDA with additional measurements of CBF using contrast enhanced NIRS to calibrate with DCS measurements. Whilst there was good correlation with the ICG flow tracer measurements of CBF, the continuous and non-invasive monitoring provided by DCS in the neonatal population has obvious advantages over methods requiring injections of exogenous tracers that provide few measurements. A single study by Dehaes et al. utilizing the hybrid FDNIRS-DCS device in newborns with NE has been described earlier (69) and represents an exciting direction in optical instrumentation with hybrid platforms offering the ability to simultaneously measure important parameters such as blood flow and metabolism more directly in this disease. Further hybrid examples will be discussed in new and emerging technology.

## New Optical Monitors and Future Directions

### New Commercial Monitors

Newer commercial devices have been developed for use particularly in the preterm population in an effort to identify variations that may precede preterm brain injury. The BabyLux (TRS NIRS+DCS) monitor has been developed to measure cerebral blood flow and absolute cerebral tissue oxygen saturation in the preterm population. The technology has integrated DCS with time-resolved (TRS) NIRS which is an evolution of CW-NIRS and offers absolute hemoglobin concentrations and tissue saturations (108). Oxyprem is a NIRS based cerebral oximeter which utilizes a self-correcting algorithm to cancel multiple in-homogeneities beneath the light source or detector and increase robustness to motion artifacts thus providing a more accurate measurement of absolute cerebral tissue oxygen saturation (StO_2_) (78).

### Emerging Optical Research Instruments

Several research groups are pushing the boundaries of current developments in new optical systems to monitor a wider range of pathophysiological information in NE in real time. Metaox is a new commercial FDNIRS-DCS hybrid instrument. Measuring arterial oxygen saturation from pulse oximeters, quantitative measurements of Hb concentration and oxygenation using FD-NIRS and CBF_i_ using DCS, the instrument derives the cerebral oxygen metabolism index (CMRO_2i_) (89, 109). Diffuse Optical Tomography (DOT) is an extension of functional NIRS (fNIRS) that combines hemodynamic information from dense optical sensor arrays and using image reconstruction techniques, can provide images of the hemodynamic correlates to neural function that are comparable to those produced by functional MR imaging (110). The UCL group in London is currently using a new dual optical platform combining BNIRS and DCS, developed in collaboration with the researchers in ICFO Barcelona, for direct measurements of mitochondrial oxidative metabolism, cerebral oxygenation and CBF_i_ in NE. This instrument has the potential to further improve our understanding of pathophysiology following perinatal hypoxic ischaemic injury. Rajaram et al. have used a similar combined BNIRS and DCS platform in a pre-clinical piglet experiment to provide information regarding clinically significant hemodynamic events prior to the onset of brain injury, which holds great potential for use particularly in the preterm neonatal population (70). The same group have also used this dual platform to monitor cerebral hemodynamics and metabolism in a cohort of neonatal patients with post-haemorrhagic ventricular dilatation undergoing ventricular tap (111). In the NE population, this dual monitoring will pave the way for further work utilizing the combined measurements of metabolic and autoregulatory disturbances in order to develop a robust cot side biomarker of injury.

## Limitations

Despite being well-suited to the neonatal population, current NIRS technology still poses some limitations. Hair, scalp oedema, movement artifact and strong light from additional sources all have the potential to cause interference with NIRS monitoring and must be carefully accounted for. Following brain insult, pathophysiological changes may show regional variation and although NIRS technology achieves good tissue penetration in neonates, spatial resolution can be limited. Research groups are currently applying other forms of NIRS technology using 3D image reconstruction such as diffuse optical tomography (DOT) which may better demonstrate spatial variations in NIRS parameters across the cortex (110, 112). Manufacturers use a range of different techniques and algorithms, most unpublished, to determine absolute cerebral tissue oxygen saturation values. Although there is good correlation between some devices, further work is needed to allow easier conversion of values from one oximeter to another and a uniform terminology is needed in order for readers to better compare findings from different studies. Various neonatal probes are also in use on the same commercial devices producing different measurements hence it is important to state the probes used with reference values for each study. There is also much variability in outcome measures between the studies described here and therefore larger cohort optical studies are needed using commonly agreed gold standard outcomes and similar methodology. Current oxCCO measurements in the hospital provide trend information of the metabolic status of the brain tissue and this trend information in combination with the continuous blood pressure measurements can derive an absolute quantification of brain metabolic autoregulation that can prognosticate infants with NE (67). Recent work in the preclinical model of HIE (61) also demonstrated that an absolute measurement of the redox state of CCO, early following hypoxic-ischaemic injury, will provide an enhanced marker of diagnosis of brain injury severity; this will need further developments in algorithms and instrumentation to deliver this in the hospital (113).

## Conclusion

The understanding of pathophysiology in NE with its biphasic and continuous evolution of injury has helped shape the type of neuromonitoring tools that are most desired by researchers and clinicians alike. A reliable neuromonitoring device should be safe and easy to use at the cot side and provide continuous monitoring that can be interpreted in real time by clinicians in the hope of directing clinical care, assessing response to neuroprotective treatments and contribute valuable prognostic information of neurological outcome. Current neuromonitoring tools such as EEG/aEEG continue to play a vital role particularly in seizure detection and management but their ability to prognosticate outcome early on in disease progression has been ameliorated with the introduction of therapeutic hypothermia. MRI/MRS, whilst being the current gold standard technique in brain injury stratification and prognostication provides snapshot information days post potential early treatment windows. A real need has developed to advance the neuromonitoring potential in NE and optical technology is paving the way to fulfilling that need with significant advances in both the technology itself and data analysis of its outputs.

Near infrared spectroscopy has developed substantially over the past 4 decades with increasing focus in the last decade on its use in the monitoring of neonatal brain injury in intensive care. There have been significant advancements in both research and commercial devices along with the use of more complex signal processing techniques which allow the dynamic relationships between multiple measurements to be characterized to form real time biomarkers of injury as they are related to gold standard outcome measures. Cerebral oximetry is undergoing improvements in both the incorporated NIRS modes and the algorithms used in order to make measurements of cerebral tissue oxygen saturation more accurate and reduce known interference. The evolution of broadband NIRS has meant that a long sought after brain tissue biomarker, Cytochrome C oxidase, can now be measured to more directly monitor the oxygen utilization of mitochondria in the injured brain. The all-optical measurement of CMRO_2i_ is also proving to be a useful measurement of brain metabolic state. Another key factor in the pathophysiology underpinning NE, altered cerebral autoregulation, has been characterized so far with NIRS monitoring by the development of advanced signal processing techniques and the formation of reactivity indices which have shown promise as potential biomarkers of injury. These reactivity indices provide further potential windows of opportunity for improving outcomes by optimizing cardiovascular stability and MABP. A further optical technology developed more recently, diffuse correlation spectroscopy, will have a major impact on the optical neuromonitoring potential in NE due to its ability to directly measure microvascular blood flow allowing more accurate calculation of autoregulatory changes and CMRO_2i_. When used in combination with NIRS platforms our ability to monitor brain health following injury is further increased.

The ultimate goal in neuromonitoring in NE is to identify a robust and accurate cot side biomarker, one that can be employed by neonatologists within hours of birth to track injury evolution in real time, identify eligible infants for appropriate neuroprotective treatments and assess their response, accurately stratify injury severity and provide early prognostication of outcome to assist in directing care and improving outcomes. Optical monitoring technology has shown much promise in achieving this goal and ongoing work in this area hopes to ensure a suitable biomarker of cerebral well-being is on the horizon.

## Author Contributions

KH-J and SM conceptualized the review and completed the first draft. FL, IT, and NR contributed to further revision and final version of the submitted manuscript. All authors contributed to the article and approved the submitted version.

## Conflict of Interest

The authors declare that the research was conducted in the absence of any commercial or financial relationships that could be construed as a potential conflict of interest.
